# Metabolomics Analysis of Splenic CD19^+^ B Cells in Mice Chronically Infected With *Echinococcus granulosus sensu lato* Protoscoleces

**DOI:** 10.3389/fvets.2021.718743

**Published:** 2021-09-06

**Authors:** Yuxin Guo, Daxiang Xu, Zheng Fang, Shiping Xu, Jiaxi Liu, Zixuan Xu, Jikai Zhou, Zhenzhen Bu, Yingyi Zhao, Jingmei He, Xiaoying Yang, Wei Pan, Yujuan Shen, Fenfen Sun

**Affiliations:** ^1^Jiangsu Key Laboratory of Immunity and Metabolism, Department of Pathogen Biology and Immunology, Xuzhou Medical University, Xuzhou, China; ^2^The First Clinical Medical College, Xuzhou Medical University, Xuzhou, China; ^3^National Experimental Teaching Demonstration Center of Basic Medicine, Xuzhou Medical University, Xuzhou, China; ^4^National Institute of Parasitic Diseases, Chinese Center for Disease Control and Prevention, Chinese Center for Tropical Diseases Research, NHC Key Laboratory of Parasite and Vector Biology, WHO Collaborating Centre for Tropical Diseases, National Center for International Research on Tropical Diseases, Shanghai, China

**Keywords:** *Echinococcus granulosus*, B cell, metabolomics, immunometabolism, metabolite, metabolic reprogramming

## Abstract

**Background:** The larval stages of *Echinococcus granulosus sensu lato* (*E. granulosus s.l*) infection can alter B cell function and affect host anti-infective immunity, but the underlying mechanism remains unclear. The newly emerging immunometabolism highlights that several metabolites are key factors in determining the fate of immune cells, which provides a new insight for exploring how larval *E. granulosus s.l*. infection remodels B cell function. This study investigated the metabolomic profiles of B cells in mice infected with *E. granulosus s.l*. protoscoleces (PSC).

**Results:**Total CD19^+^ B cells, purified from the spleen of infected mice, showed significantly increased production of IL-6, TNF-α, and IL-10 after exposure to LPS *in vitro*. Moreover, the mRNA expression of metabolism related enzymes in B cells was remarkably disordered post infection. In addition, differential metabolites were identified in B cells after infection. There were 340 differential metabolites (83 upregulated and 257 downregulated metabolites) identified in the positive ion model, and 216 differential metabolites (97 upregulated and 119 downregulated metabolites) identified in the negative ion mode. Among these, 64 differential metabolites were annotated and involved in 68 metabolic pathways, including thyroid hormone synthesis, the metabolic processes of glutathione, fructose, mannose, and glycerophospholipid. Furthermore, several differential metabolites such as glutathione, taurine, and inosine were validated to regulate the cytokine production in LPS stimulated B cells.

**Conclusion:**Infection with the larval *E. granulosus s.l*. causes metabolic reprogramming in the intrinsic B cells of mice, which provides the first evidence for understanding the role and mechanism of B cells in parasite anti-infective immunity from the viewpoint of immunometabolism.

## Introduction

Cystic echinococcosis (CE) is a zoonosis caused by the larval stages of *Echinococcus granulosus sensu lato* (*E. granulosus s.l*.) and is one of the neglected tropical diseases recognized by the World Health Organization (1, 2). The larvae of *E. granulosus s.l*. is the most harmful to the human body, which usually leads to a pathology through the compression of the host organ and severe complications carrying the risks of anaphylactic shock. Moreover, the protoscoleces (PSCs) released from spontaneous or trauma-induced cyst rupture can grow to a new cyst in *vivo*, result in a serious secondary CE. The parasite is distributed worldwide (3) and causes heavy economic losses globally (4). Notably, *E. granulosus s.l*. can infect hosts and go unnoticed for several decades, as it has evolved immune subversive strategies to resist host anti-infective immunity. Therefore, understanding the molecular mechanism of these strategies is beneficial for identifying the host-parasite interplay and developing novel immunologic intervention strategies for preventing and controlling CE.

Evidence shows that the immune response to the larval *E. granulosus s.l*. infection involves a variety of immune cells, forming a complex immune regulatory network (5, 6). Parasitic factors such as excretory secretion products can affect the activation of antigen-presenting cells such as dendritic cells and macrophages, induce Th2 dominated immune response, and inhibit Th1 and Th17 immune responses, thereby lowering the host anti-infection immunity (7, 8). Furthermore, larval *E. granulosus s.l*. infection can induce the accumulation of regulatory T cells (Tregs), regulatory B cells (Bregs), and myeloid-derived suppressor cells, building an anti-inflammatory host environment in mice (9–11). In addition, there is evidence of inflammatory and granulomatous reactions against *E. granulosus* sensu stricto metacestodes in naturally infected hosts, such as cattle and sheep, which are related to the non-fertility of CE cysts (12, 13). Taken together, these immune responses contribute to the long-term survival of the parasite and induce immunopathology in the host.

B cells are capable of producing antibodies and are involved in autoimmunity, cancer, and infections as regulatory cells (14). In recent years, B cells have also been shown to play a vital role in parasitic infections and immunity. It was reported that B cells producing IL-10 are essential for inhibiting type I hypersensitivity in BALB/c mice infected with *Leishmania major* (15). Moreover, this subgroup was also reported to accumulate in the sera of patients with *Schistosoma mansoni* and *Echinococcus multilocularis* infections (16, 17). In addition, our previous studies showed that larval *E. granulosus s.l*. infection and its excretory-secretory products (ESPs) can stimulate IL-10 production in splenic B cells via TLR-2 signaling (18, 19). However, the underlying molecular mechanisms remain unclear.

Immunometabolism, the interplay between immunological and metabolic processes, has gained interest as an emerging field of investigation in recent years (17). Metabolic reprogramming has been demonstrated to be a key prerequisite for determining the differentiation and effects of immune cells (20–23), which provides a novel insight into the immunoregulatory mechanism of B cells in the context of parasitic infections. Recently, there has been evidence that changes in metabolic composition directly contribute to altered B cell function (24). Glucose, palmitic acid, amino acid homocysteine, and short chain fatty acids have all been shown to directly impact B cell fate and function (25–28). It has also been reported that B cells undergo metabolic reprogramming, mainly relying on aerobic glycolysis and glucose transporter-dependent metabolic pathways to support antibody production (29). Surprisingly, cholesterol metabolism drives IL-10 production in Bregs through the provision of geranylgeranyl pyrophosphate (30). In summary, these findings have broadened our understanding of how metabolic events determine B cell function. However, it is still unknown if parasitic infection triggers metabolic reprogramming to determine the host anti-infectious immunity in B cells.

This study investigated the metabolomic profiles of splenic B cells in a secondary CE mouse model. The results showed that parasitic infection remodels B cell function, along with dramatic intrinsic metabolic reprogramming. Overall, our findings provide the first evidence for understanding the role and mechanism of B cells against secondary CE infections in mice.

## Materials and Methods

### Mice, Parasites, Infection

C57BL/6J female mice (aged 6–8 weeks) were obtained from the Shanghai Laboratory Animal Center (Shanghai, China) and bred in the Experimental Animal Center of Xuzhou Medical University. The mice were randomly divided into two groups with fifteen mice in each group (*n* = 15): *E. granulosus s.l* group (model group, M) and control group (control check, CK). The PSCs of *E. granulosus s.l*. (EgPSCs) were obtained by puncturing the fertile sheep hydatid cysts under aseptic conditions according to the protocols detailed in Carmena et al. (31, 32), and the method for the establishment of PSC infected mice was mentioned in our previous studies (11). At the end of the experiment, 30 mice were euthanized by intraperitoneal injection of 0.2 ml 4% sodium pentobarbital anesthesia solution. All animal procedures were approved by the Laboratory Animal Welfare and Ethics Committee of Xuzhou Medical University, China.

### B Cell Isolation

CD19^+^B cells from the spleens of control or infected mice were sorted negatively using a mouse CD19^+^ B cell isolation kit (Miltenyi, Bergisch Gladbach, Germany); the cell purity identified by flow cytometry was routinely >90%. The isolated B cells were further used for *in vitro* cultivation and metabolomic analyses.

### Quantitative Real-Time PCR

Total RNA was extracted from CD19^+^B cells using the TRIzol reagent, and cDNA was synthesized from the RNA using the PrimeScript™ RT Master Mix. Quantitative PCR analyses were performed in a LightCycler® 480II detection system (Roche Applied Science, Penzberg, Germany) under the following thermal cycling conditions: one cycle of 5 min denaturation at 95°C, followed by 30 s at 95°C, 30 s at 60°C, and 30 s at 72°C for 45 cycles using the primers listed in Supplementary Table 1. The mRNA levels of specific genes were normalized to β-actin mRNA levels.

### *In vitro* Cultivation of B Cells

Purified B cells from the spleens of control and infected mice were cultured in 24-well plates (2 × 10^6^ cells/well) in the presence or absence of LPS (10 μg/ml). Blank medium without fetal bovine serum served as the vehicle control. After 24 h of culture, the supernatants were collected for cytokine analysis. Alternatively, several metabolites such as glutathione (8 mM, Bidepharm, China), taurine (2.5 mM, Bidepharm, China), and inosine (5 mM, Bidepharm, China) were added into the sorted B cells for 2 h followed by 24 h stimulation with LPS (10 μg/ml). Then the cytokine levels in the supernatants were determined.

### Cytokine Analysis

The levels of TNF-α, IL-6, and IL-10 in the culture supernatants were detected using mouse IL-6, TNF-α, and IL-10 ELISA Ready-SET-Go! Kit (eBioscience, USA), according to the manufacturer's recommendations. Cytokine concentrations were calculated using the standard curves.

### Metabolite Extraction and LC-MS/MS Analysis

Approximately 5 × 10^6^ splenic B cells were sorted for each sample. Each group contained six samples from six individual controls or infected mice. LC-MS/MS analysis was performed by Guangzhou Gene Denovo Co. (Guangzhou, China). After the addition of 1,000 μl of extract solvent (acetonitrile-methanol-water, 2:2:1, containing internal standard), the samples were vortexed for 30 s, homogenized at 45 Hz for 4 min, and sonicated for 5 min in an ice-water bath. The homogenization and sonication were repeated three times, followed by incubation at −20°C for 1 h and centrifugation at 12,000 rpm and 4°C for 15 min. The resulting supernatants were transferred to LC-MS vials and stored at −80°C until UHPLC-QE orbitrap/MS analysis. The quality control (QC) sample was prepared by mixing an equal aliquot of the supernatant from all samples.

LC-MS/MS analyses were performed using a UHPLC system (1290, Agilent Technologies) with a UPLC HSS T3 column (2.1 × 100 mm, 1.8 μm) coupled to Q Exactive (Orbitrap MS, Thermo). The mobile phase A was 0.1% formic acid in water for positive, 5 mmol/L ammonium acetate in water for negative, while mobile phase B was acetonitrile. The elution gradient was set as follows: 0 min, 1% B; 1 min, 1% B; 8 min, 99% B; 10 min, 99% B; 10.1 min, 1% B; 12 min, 1% B. The flow rate was 0.5 ml/min. The injection volume was 2 μl. The QE mass spectrometer was used for its ability to acquire MS/MS spectra on an information-dependent basis (IDA) during an LC/MS experiment. In this mode, the acquisition software (Xcalibur 4.0.27, Thermo) continuously evaluates the full scan survey MS data as it collects and triggers the acquisition of MS/MS spectra depending on preselected criteria. The ESI source conditions were set as follows: sheath gas flow rate, 45 Arb; Aux gas flow rate, 15 Arb; capillary temperature, 320°C; full ms resolution 70,000; MS/MS resolution 17,500; collision energy, 20/40/60 eV in the NCE model; spray voltage, 3.8 kV (positive) or −3.1 kV (negative).

### Data Reprocessing and Annotation

MS raw data files were converted to the mzML format using ProteoWizard and processed by R package XCMS (version 3.2), including retention time (RT) alignment, peak detection, and peak matching. The data were then filtered by the following criterion: the number of samples containing a metabolite were <50% of all sample numbers in a group (QC were also taken as a group). Afterwards, normalization to an internal standard (33) for each sample was performed. The missing values were then replaced by half of the minimum value found in the dataset by default (34). The preprocessing results generated a data matrix that consisted of the RT, mass-to-charge ratio (m/z) values, and peak intensity. OSI-SMMS (version 1.0, Dalian Chem Data Solution Information Technology Co. Ltd.) was used for peak annotation after data processing using an in-house MS/MS database.

### Multivariate Statistical Analysis

Principal component analysis (PCA), partial least squares discrimination analysis (PLS-DA), and orthogonal partial least squares discriminant analysis (OPLS-DA) were performed after data preprocessing. Principal component analysis was applied to all samples, and PLS-DA and OPLS-DA were applied to control groups using R package models (http://www.r-project.org/).

### Differential Metabolites Analysis

Fold change (FC) and *T*-test of the data were used to detect and identify differential metabolites between the control and infected groups (35), and univariate analysis was used to determine the changes in metabolites between the two groups, so as to screen out the significant differences in metabolites. Those with a *P-*value of < 0.05, and |FC| ≥1.5, were considered differential metabolites between the two groups.

### Kyoto Encyclopedia of Genes and Genomes Pathway Analysis

Kyoto Encyclopedia of Genes and Genomes (KEGG) is the major public pathway-related database that includes not only genes but also metabolites (36). Metabolites were mapped to KEGG metabolic pathways for pathway and enrichment analyses. Pathway enrichment analysis identified significantly enriched metabolic pathways or signal transduction pathways in the differential metabolites compared to the whole background. The calculated *P-*value was subjected to FDR correction, with FDR ≤ 0.05, as a threshold. Pathways meeting this condition were defined as significantly enriched pathways in the differential metabolites.

### Statistical Analysis

Data were analyzed using GraphPad Prism software 8.0 and are presented as mean ± standard error of the mean. Statistical significance was determined using the unpaired two-tailed Student's *t*-test for a single variable and one-way analysis of variance (ANOVA) followed by the *post hoc* Tukey test for multiple comparisons. Values with *p* < 0.05 were considered statistically significant.

## Results

### Changes to the Cytokine Profiles in Splenic B Cells Post EgPSC Infection

**Figure 1:**
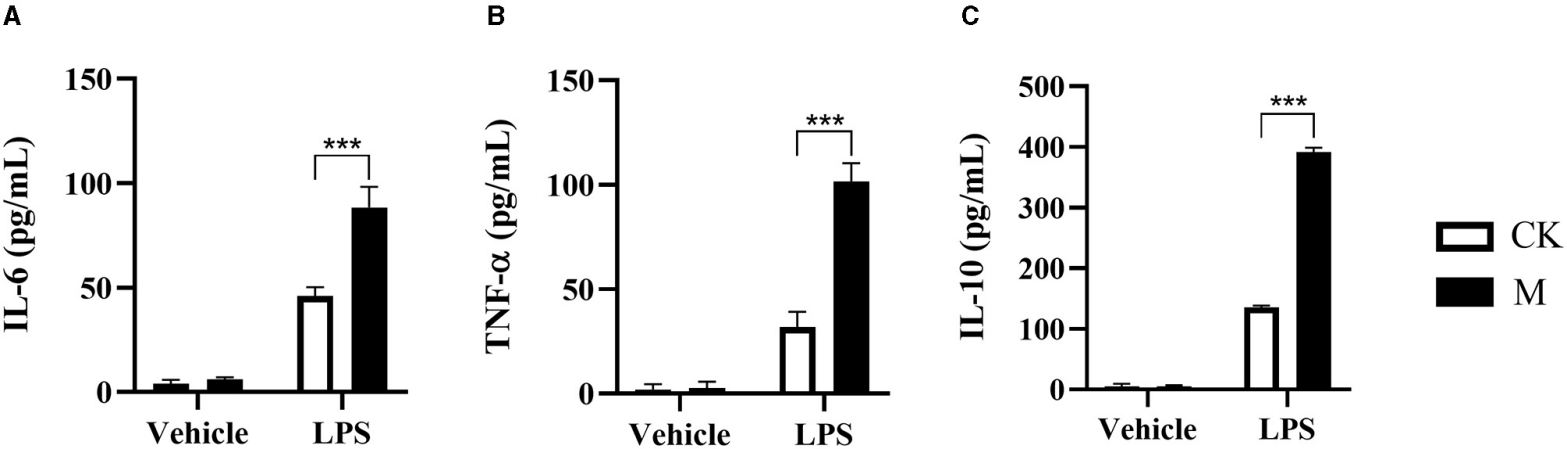
Changes to cytokine in the splenic B cells post EgPSC infection. The CD19^+^B cells were isolated from the spleens of control or EgPSC infected mice and cultured with vehicle or LPS (10 μg/ml) for 24 h. The cytokine level in the culture supernatants was detected by ELISA. **(A)** IL-6, **(B)** TNF-α, and **(C)** IL-10. Values are mean ± standard error of means. *n* = 4 mice per group. CK, control check group; M, model group. (Student's *t*-test: ***P < 0.001).

B cells are one of the contributors to anti-host immunity (14). Our previous studies have shown that EgPSC infection or its derived ESPs can elevate IL-10 production in splenic B cells and increase the percentage of Bregs *in vivo* and *in vitro*, respectively (11, 18). However, the mechanism by which the parasite affects B cell function remains unclear. To further explore the effects of secondary CE infection on B cell function, splenic CD19^+^B cells were sorted from control and infected mice and cultured with a vehicle or LPS (10 μg/ml) for 24 h. LPS, a TLR ligand known as a murine B cell activator, was used as a positive indicator that promotes pathogen invasion and induces an inflammatory response in B cells. Afterwards, the cytokine levels in the culture supernatants were measured. As shown in Figure 1, there were almost no detectable cytokines in the supernatants of vehicle control or infected splenic B cells. However, in the presence of LPS, infected B cells produced significantly higher levels of IL-6, TNF-α, and IL-10 than those in control B cells (all *P* < 0.001). The tendency of cytokine production was in line with the results observed in parasite-infected peritoneal B cells (37). Collectively, these results indicate that EgPSC infection activates B cells.

### mRNA Expression of Metabolism Related Enzymes in the Splenic B Cells Post EgPSC Infection

**Figure 2:**
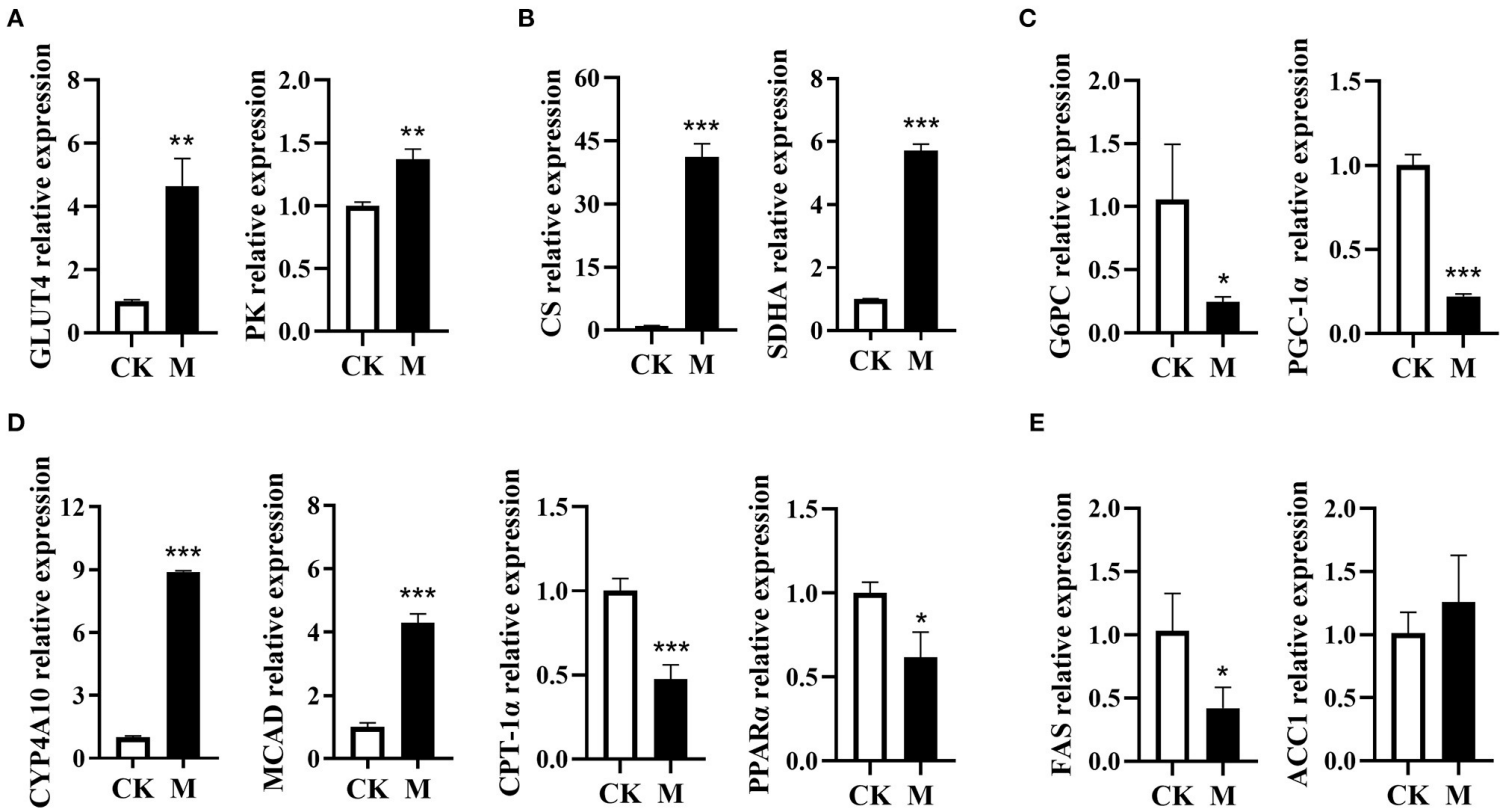
Expression profiling of genes encode the enzymes in metabolic pathways in the splenic B cells post EgPSC infection. B cells were obtained from the spleens of control or EgPSC infected mice, and the mRNA expression of the genes encode the enzymes in metabolic pathways was characterized by RT-PCR. **(A)** GLUT4 and PK, **(B)** CS and SDHA, **(C)** G6PC and PGC-1α, **(D)** CYP4A10, MCAD, CPT-1α, and PPARα, and **(E)** FAS and ACC1. Values are mean ± standard error of means. n = 4-5 mice per group. CK, control check group; M, model group; GLUT4, glucose transporter 4; PK, pyruvate kinase; CS, citrate synthase; SDHA, succinate dehydrogenase complex subunit A; G6PC, glucose-6-phosphatase; MCAD, medium-chain acyl-CoA dehydrogenase; CYP4A10, cytochrome P450 proteins 4; CPT-1α, carnitine palmitoyl transferase 1a; PPARα, peroxisome proliferator–activated receptor α; PGC-1α, peroxisome proliferator–activated receptor-γ coactivator1α; FAS, fatty acid synthase; ACC1, acetyl coenzyme A carboxylase 1 (Student's *t*-test: *P < 0.05, **P < 0.01, ***P < 0.001).

B cell activation is orchestrated by a complex network of intracellular signaling pathways and transcription factors. To meet the energetic and biosynthetic demands of protein synthesis and cell division, signal transduction pathways reshape the metabolic profile of activated B cells (24). To characterize the metabolic events in B cell differentiation induced by EgPSC infection, this study further detected the mRNA expression of genes that encode the rate-limiting enzymes in bioenergy pathways. In comparison to control B cells, the infected B cells exhibited higher mRNA expression of the genes (glucose transporter 4, GLUT4; pyruvate kinase, PK) associated with glycolysis (both *P* < 0.01, Figure 2A). Moreover, the mRNA expression of citrate synthase (CS) and succinate dehydrogenase subunit A (SDHA), two key enzymes in the tricarboxylic acid cycle (TCA), was found to be distinctly higher post infection (both *P* < 0.001, Figure 2B). Nevertheless, the mRNA expression of glucose-6-phosphatase (G6PC) associated with gluconeogenesis was significantly downregulated following infection (*P* < 0.05, Figure 2C).

In contrast, the mRNA expression of fatty acid oxidation (FAO)-related genes (cytochrome P450 proteins 4A10, CYP4A10; medium-chain acyl-CoA dehydrogenase, MCAD) in infected B cells was significantly upregulated (both *P* < 0.001, Figure 2D). However, the expression of FAO-associated genes PPAR-α (peroxisome proliferator-activated receptor alpha) and CPT-1α (carnitine palmitoyl transferase 1α) was downregulated (*P* < 0.05, Figure 2D), which may be attributed to the downregulation of peroxisome proliferator-activated receptor-γ coactivator 1α (PGC-1α) (38). In addition, the expression of fatty acid synthase (FAS) in lipogenesis was downregulated, while the lipogenic gene acetyl coenzyme A carboxylase 1 (ACC1), had no obvious alteration after infection (*P* < 0.05, Figure 2E). These results suggest that EgPSC infection may induce a complex glucolipid metabolic reprogramming network in splenic B cells.

### Identification of Differential Metabolites in the Splenic B Cells in Mice Post EgPSC Infection

**Figure 3:**
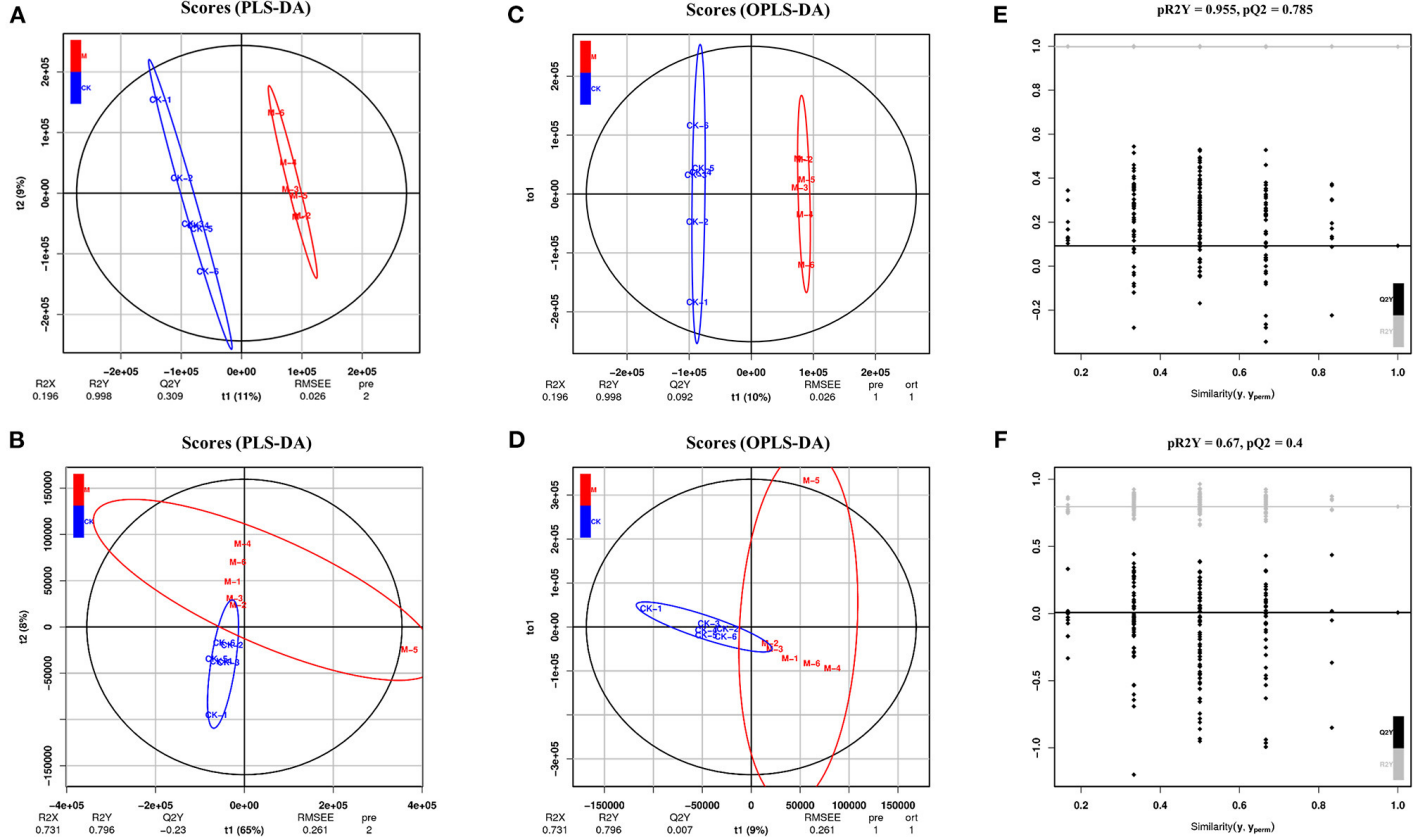
Multivariate statistical analysis in positive and negative ion mode. **(A)** PLS-DA score charts in positive ion mode. **(B)** PLS-DA score charts in negative mode. **(C)** OPLS-DA score charts in positive ion mode. **(D)** OPLS-DA score charts in negative ion mode. **(E)** OPLS-DA model validation charts in positive ion mode. **(F)** OPLS-DA model validation charts in negative ion mode. *n* = 5–6 mice per group. CK, control check; M, model group [All R2′ and Q2′ points from left to right were lower than rightmost two points (*x* = 1.0) (R2 and Q2 of the original model)]; this indicated that the model was robust and reliable without overfitting.

Emerging evidence suggests that shifts in available fuel sources and intracellular metabolite concentrations profoundly affect cell fate decisions (39). This study further characterized the differential metabolites in the splenic B cells of mice post-infection using LC-MS/MS analysis. For a preliminary visualization of differences between samples from the infected and control groups, PCA analysis was carried out using an unsupervised dimensionality reduction method. Partial least squares discrimination analysis is a supervised dimensionality reduction method in which class memberships are coded in matrix form into Y to better distinguish the metabolomic profile of the two group B cells by screening variables correlated to class memberships. The point dispersion trend of positive and negative ions in the two B cell subgroups was obvious (Figures 3A,B), indicating that there were differences in metabolites, and that the established model was stable and reliable.

To obtain these altered metabolites, the control and infected B cells were selected for OPLS-DA. There was obvious spatial separation between the two groups (Figures 3C,D). To further prove the reliability of the model, the permutation order of classification variable Y was randomly changed by a permutation test, which established a value 200 times that of the OPLS-DA model. The two rightmost points (*x* = 1.0) are R2 and Q2 of the original model, and all the points on the left are R2′ and Q2′ of the model after Y replacement. If all the R2′ and Q2′ are smaller than the original R2 and Q2 (Figures 3E,F), then the model is stable and reliable without overfitting.

**Figure 4:**
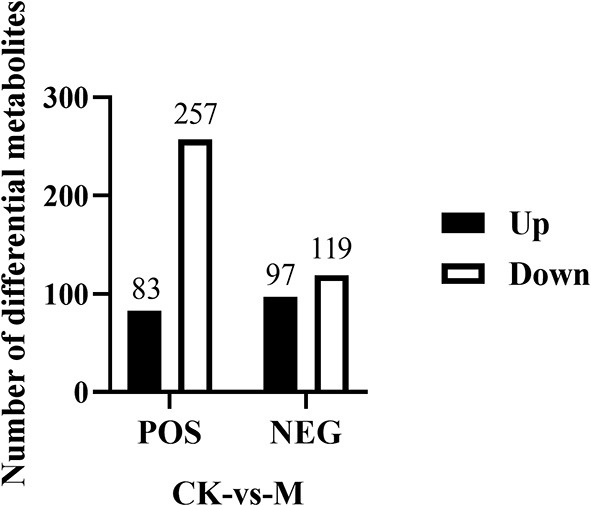
Number of differential metabolites identified in the splenic B cells post EgPSC infection. Those metabolites with a *P*-value of *T*-test < 0.05 and |FC| ≥ 1.5 were considered differential metabolites between control and model groups. *n* = 5–6 mice per group. CK, control check; M, model group.

Fold change and *T*-test as univariate statistical analysis were combined to screen for significantly different metabolites between the comparison groups. Those with a *P*-value of < 0.05, and |FC| ≥1.5, were considered differential metabolites. The results showed that in positive ion mode, a total of 340 differential metabolites were chosen (83 upregulated and 257 downregulated metabolites), while 216 differential metabolites were selected in negative ion mode (97 upregulated and 119 downregulated metabolites) (Figure 4). These differential metabolites may provide new clues for understanding the role of intracellular metabolites in the complex regulation of B cell differentiation and function.

### KEGG Pathway Analysis of Differential Metabolites of the Splenic B Cells Post EgPSC Infection

**Figure 5:**
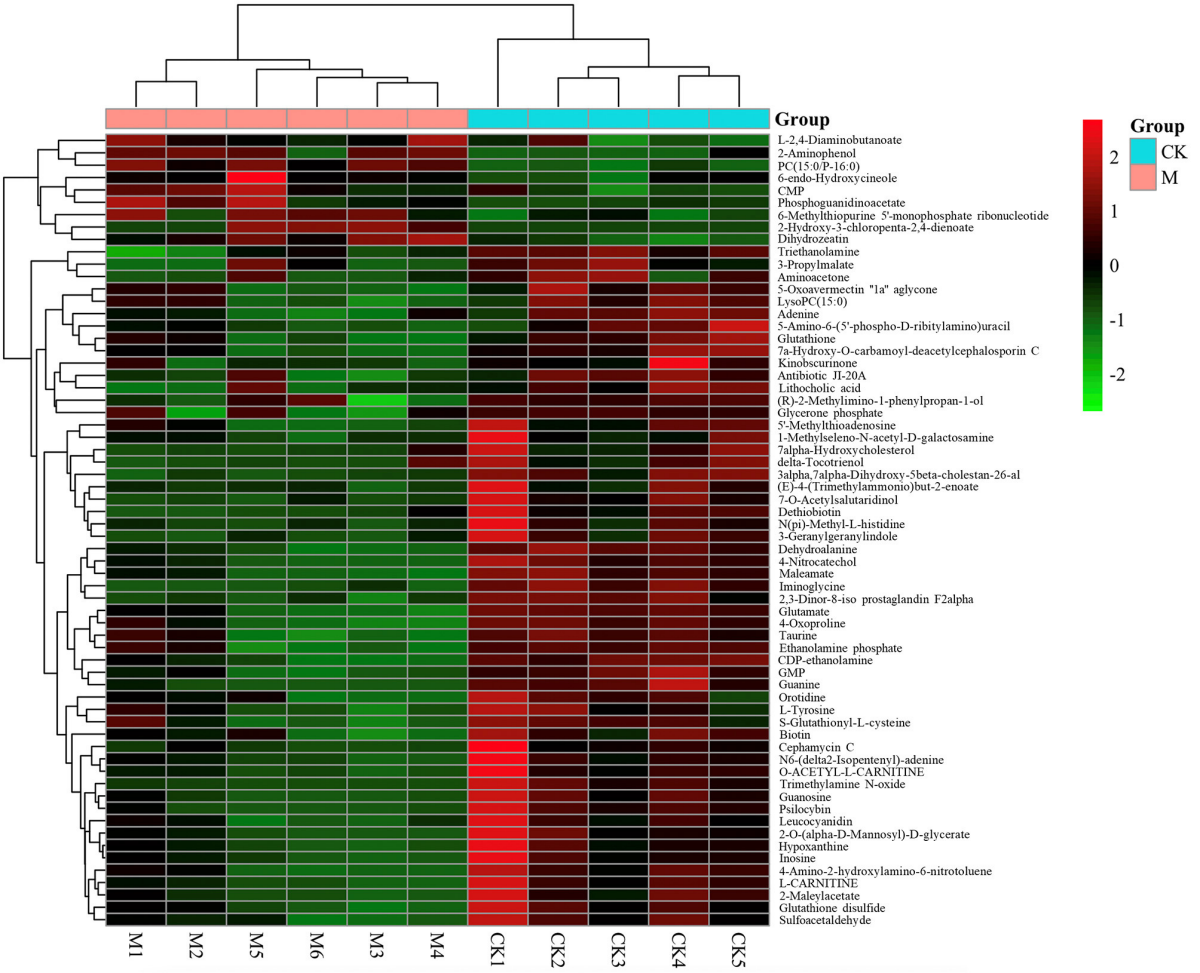
Heat-map of differential metabolites identified between model and control group by KEGG pathways. *n* = 5–6 mice per group. Rows: differential metabolites; columns: samples. The color of each small square represents the level of metabolite expression. Red: highest; green: lowest; black; mean.

**Figure 6:**
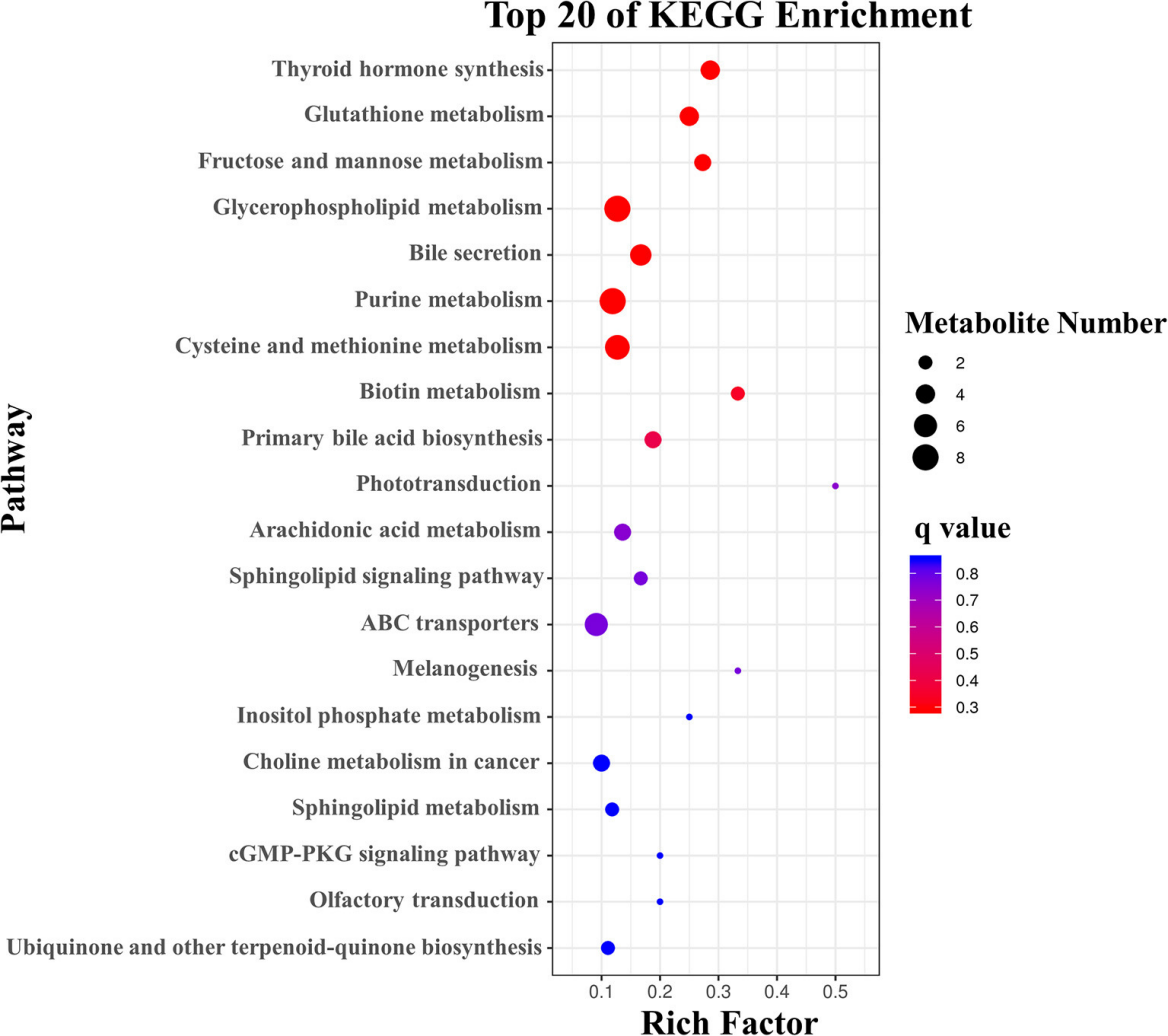
KEGG analysis of the differential metabolites with the top 20 enrichment scores.

To further assess the significantly different metabolites associated with B cell differentiation and function and confirm that the detected metabolites represent the preservation of a wide range of biochemical processes, their pathways and molecular interactions were then predicted by KEGG pathway enrichment analysis. After filtering the metabolites with unknown pathways, 64 significantly different metabolites (Figure 5) were screened for further analyses and their associated details were shown in Supplementary Table 2 These differential metabolites were involved in 68 metabolic pathways. Compared with control cells, the major metabolic pathways differed in thyroid hormone synthesis, glutathione metabolism, fructose, and mannose metabolism, glycerophospholipid metabolism, bile secretion, purine metabolism, cysteine and methionine metabolism, and biotin metabolism (Figure 6). It is reported that glutathione is essential for maintaining T cell inflammatory responses (40). Moreover, bile acid metabolites (distinct derivatives of lithocholic acid) have been demonstrated to direct Th17 and Treg cell differentiation (41). Therefore, these identified differential metabolites may be the key to controlling B cell differentiation and function through reprogramming metabolic flux.

### Functional Validation of Differential Metabolites on Splenic B Cells Immune Responses

**Figure 7:**
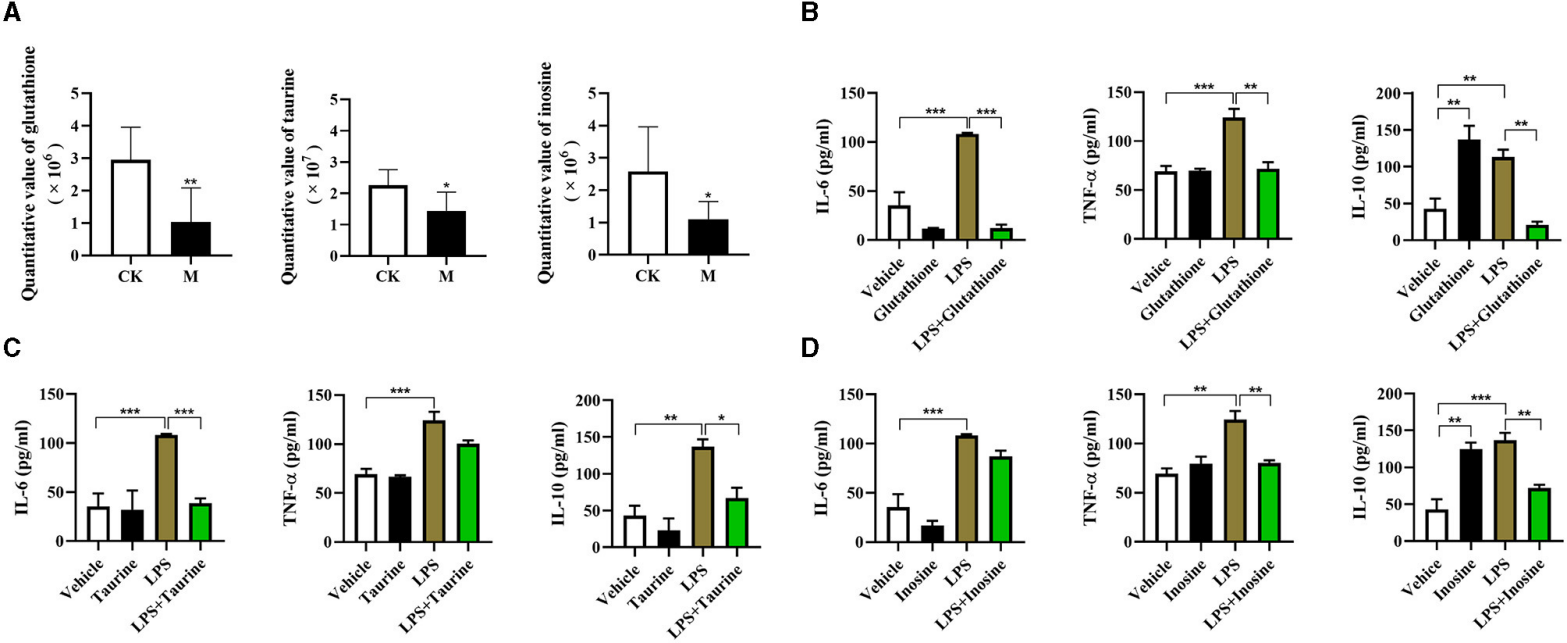
Functional validation of differential metabolites on immune responses of splenic B cells. Glutathione (8 mM), taurine (2.5 mM) and inosine (5 mM) were respectively added into the sorted B cells for 2 h followed by 24 h stimulation with LPS (10 μg/ml), and the cytokine levels in the supernatants were determined using ELISA. **(A)** The quantitative value of glutathione, taurine, and inosine determined by LC/LC-MS; **(B)** IL-6, TNF-α, and IL-10 stimulated by glutathione; **(C)** IL-6, TNF-α, and IL-10 stimulated by taurine; **(D)** IL-6, TNF-α, and IL-10 stimulated by inosine. Values are mean ± standard error of means. CK, control check; M, model group (One-way ANOVA: *P < 0.05, **P < 0.01, ***P < 0.001).

Previous studies have showed that glutathione, taurine, and inosine have anti-inflammatory effect in macrophages (42–45), suggesting that these metabolites have potential immunomodulatory functions. However, their effects on B cell immune responses are still unclear. We found a significant lower of the quantitative value of glutathione, taurine, and inosine in the splenic B cells from the infected mice (all *P* < 0.05, Figure 7A). To validate the function of differential metabolites identified, three commercially available substitutes of glutathione, taurine, and inosine were, respectively, added into the sorted normal total splenic B cells for 2 h followed by 24 h stimulation with LPS, and the cytokine levels in the supernatants were determined using ELISA. Supplementation of glutathione reduced IL-6, TNF-α, and IL-10 production in LPS stimulated B cells (*P* < 0.01, Figure 7B). Interestingly, a significant increase of IL-10 production was observed in glutathione treated B cells (*P* < 0.01, Figure 7B). The IL-6 and IL-10 production, but not TNF-α, in LPS stimulated B cells was significantly decreased after exposure to Taurine (*P* < 0.05, Figure 7C). Inosine treatment decreased the levels of TNF-α and IL-10, but not IL-6 in LPS stimulated B cells (*P* < 0.01, Figure 7D). These results suggest that these metabolites can remodel the immune profile of B cells.

## Discussion

This study showed that the PSCs of *E. granulosus s.l*. infection remodels the function of splenic B cells in mice. This is characterized by the increased production of both pro-inflammatory and anti-inflammatory cytokines along with the altered expression of the genes encoding the enzymes in metabolic pathways. Moreover, 64 differential metabolites were identified in B cells post-infection and were involved in 68 metabolic pathways. In addition, several differential metabolites were shown to have anti-inflammatory function in LPS stimulated B cells. These results suggest that parasitic infection induces metabolic reprogramming in B cells. To the best of our knowledge, this study is the first to investigate metabolic reprogramming in B cells in mice infected with the larval *E. granulosus s.l*., which may provide an immunometabolic perspective for understanding the key events that trigger B cell differentiation in the context of parasitic infection.

The larval *E. granulosus s.l*. has evolved sophisticated strategies to escape host immune responses, mainly by manipulating and directing this immune response toward anergy or tolerance (1). Our previous studies have shown that the induction of Bregs with the phenotype of CD19^+^CD1d^hi^CD5^+^ post parasite infection contributes to immune evasion (11, 18). In the present study, we further showed that both the production of pro-inflammatory cytokines (TNF-α and IL-6) and anti-inflammatory cytokine (IL-10) were significantly elevated in the splenic B cells of infected mice after exposure to LPS. It is observed that the B cells producing cytokines are undetectable both in infected and control mice (Figure 1). Thus, activation of B cells by LPS can not only help highlight the level changes of these cytokines, but also reflect the capability of host response against another pathogen infection and second infection of the parasite itself. Our results confirmed that larval *E. granulosus s.l*. infection can alter the function of B cells. Notably, the level of IL-10 in infected B cells was significantly higher than that in TNF-α and IL-6. In addition, it is well-known that IL-10 has a strong capability to suppress immune responses (46). Our findings indicate that B cell-derived IL-10 may contribute to maintaining a compromised immune status in the host, thereby allowing the slowing growth of the parasites or their second infection.

In view of the emerging immunometabolism perspective, the function of immune cells is governed by metabolic pathways (19), which are specifically regulated by rate-limiting enzymes. For example, arginase was recently shown to promote the immune evasion of *E. granulosus* in mice by inhibiting the expression of the T cell receptor CD3ζ chain (47). Our recent studies showed that the reprogramming of glucose and lipid metabolism has been linked to liver fibrosis in mice infected with *S. japonicum* (48, 49). To investigate the underlying mechanism of altered B cell function, we characterized the primary expression profiles of several key enzymes involved in the bioenergy pathways in bulk B cells post larval *E. granulosus s.l*. infection. This study showed that the mRNA levels of enzymes associated with the glycolysis and TCA pathways were significantly upregulated after infection, while the expression of lipogenic genes was obviously downregulated. However, there were some differences with regard to the enzyme expression of genes involved in FAO. MCAD catalyzes crucial steps in mitochondrial FAO, and CYP4A10 is a cytochrome P450 fatty acid hydroxylase that catalyzes the ω-hydroxylation of medium-and long-chain fatty acids and prostaglandins. They were both significantly upregulated, indicating that FAO is activated in B cells post-infection. However, other FAO-associated genes, PPAR-α and CPT-1α, were downregulated. PPAR-α and PGC-1α can elevate the expression of CPT-1α in hepatocytes (38), which may be one of the reasons for this decrease. These results suggest that metabolic reprogramming occurs in B cells. In macrophages, glycolysis and FAO have been demonstrated to determine the polarization of M1 and M2, respectively (50). The present study therefore speculated that such metabolic remodeling may be closely linked with alteration of B cell function after larval *E. granulosus s.l*. infection.

Metabolites are the most direct substances that regulate the immune response. To date, several metabolites (such as glucose, palmitic acid, amino acid homocysteine, and short chain fatty acids) have been demonstrated to play a crucial role in reprogramming B cell function (25–28). However, the profile of differential metabolites that may be associated with B cell function in *E. granulosus s.l*. infected mice has not been reported. This study mapped 64 enriched KEGG pathways, including thyroid hormone synthesis, the metabolism of glutathione, fructose, mannose, glycerophospholipid, purine, cysteine, methionine, and bile secretion. These results provide several clues for the further screening of the specific metabolite(s) that determine(s) B cell functional differentiation.

Firstly, three metabolites identified in this study can program B cell immune function *in vitro*. It has been reported that glutathione can contribute to the control of intracellular *Mycobacterium tuberculosis* infection by reducing the levels of pro-inflammatory cytokines (TNF-α, IL-6, and IL-1) (43). In line with this, the present study observed that glutathione supplementation inhibits the TNF-α and IL-6 in B cells. Moreover, we also validated the anti-inflammatory effect of inosine and taurine. Inosine has been reported to inhibit the production of the proinflammatory cytokines TNF-α, IL-1, IL-12, macrophage-inflammatory protein-1a and IFN-γ in immune stimulated macrophages (42), while taurine can inhibit the secretion of pro-inflammatory cytokines including IL-6, TNF-α, and IL-8 (44, 45). Thus, the decreased levels of the three metabolites in the splenic B cells post larval *E. granulosus s.l*. may in turn enhance the production of IL-6, TNF-α, and IL-10.

Secondly, we found several metabolites with the potential to regulate the B cell immune function. Cholesterol metabolism has been shown to participate in restricting inflammatory responses (51–53). The inhibition of HMG-CoA reductase, a key enzyme in the early step of the cholesterol metabolic pathway, has been reported to impair the ability of B cells to produce IL-10, both at the mRNA and protein levels (30). Cholesterol metabolism in regulating IL-10 production independent of phenotype that is shared across B cell populations, rather than an effect on specific populations (30). Moreover, there is evidence that δ-tocotrienols can lower serum total and LDL cholesterol levels by inhibiting HMG-CoA reductase activity (54). In this study, the production of δ-tocotrienols was decreased in splenic B cells following larval *E. granulosus s.l*. infection. Thus, δ-tocotrienols may be another key metabolite that induces B cell functional differentiation. Besides, the 7a-Hydroxycholesterol level in B cells was also decreased post infection. Accumulating evidence suggests that this metabolite participates in the inflammatory response through various pathways. It has been reported that in atherosclerosis, 7a-Hydroxycholesterol can elicit TLR6-mediated expression of IL-23 by monocytic cells via PI3K/Akt and MAPKs pathways (55), leading to inflammation via the upregulation of CCL2 and MMP-9 in macrophages (56). It also causes the enhanced transcript levels of IL-8 and the secretion of its corresponding gene product by monocytes/macrophages (57), thereby promoting the progression of atherosclerosis. In addition, a decreased level of trimethylamine N-oxide (TMAO) was observed after larval *E. granulosus s.l*. infection, and this metabolite was previously reported to promote inflammatory responses (58, 59). Lastly, there is evidence that autocrine stimulation of IL-10 is critical for the enrichment of IL-10-producing CD40^hi^CD5^+^ Bregs *in vitro* and *in vivo* (60), which may indicate that the elevated IL-10 production in larval *E. granulosus s.l*. infection may in turn enhance IL-10 secretion by B cells, thereby helping the immune evasion. However, the exact role and mechanism of these identified metabolites requires further investigation.

In fact, this study aimed to investigate the key metabolites that can guide Bregs differentiation post larval *E. granulosus s.l*. infection. However, due to the low number of Bregs in the spleen, we could not collect enough Bregs for metabolomics analysis. Therefore, bulk B cells were investigated in this study. However, as mentioned above, we identified several candidate metabolites for future studies on Bregs differentiation to gain insight into immunometabolism. Furthermore, what we have to point is that the mice model used in the study is established by abdominal infection of the PSCs, which is widely used in other groups and our previous studies (11, 18, 61). Notably, this animal model can mimic secondary CE. However, this model also has some limitations, because it cannot completely reflect the immune profiles of larval *E. granulosus s.l*. in sheep and cattle naturally infected by ingesting the eggs of the parasite (12, 13).

## Conclusion

This study showed functional alternation along with dramatic metabolic reprogramming of splenic B cells in a secondary CE mouse model. Moreover, differential metabolites were identified using metabolomic analysis. These findings provide a novel insight for clarifying the underlying mechanism of B cell functional differentiation and host anti-infective immunity induced by infection with the parasite.

## Data Availability Statement

The original contributions presented in the study are included in the article/Supplementary Material, further inquiries can be directed to the corresponding author/s.

## Ethics Statement

The animal study was reviewed and approved by Ethics Committee of Xuzhou Medical University (Xuzhou, China, SYXK (Su) 2020-0048).

## Author Contributions

FS, WP and YS: conceived and designed the experiments. YG, DX, ZF, SX, JL, and ZX: performed the experiments. JZ, ZB, YZ, and JH: analyzed the data. WP, FS, XY, and YS: contributed reagents, materials, and analysis tools. YG, DX, ZF and SX: wrote the manuscript. All authors have read and approved the manuscript.

## Conflict of Interest

The authors declare that the research was conducted in the absence of any commercial or financial relationships that could be construed as a potential conflict of interest.

## Publisher's Note

All claims expressed in this article are solely those of the authors and do not necessarily represent those of their affiliated organizations, or those of the publisher, the editors and the reviewers. Any product that may be evaluated in this article, or claim that may be made by its manufacturer, is not guaranteed or endorsed by the publisher.
