# Supplementary material for: Metabolomics Analysis of Splenic CD19+ B Cells in Mice Chronically Infected With Echinococcus granulosus sensu lato Protoscoleces
Source: Front Vet Sci. 2021 Sep 6;8:718743. doi: 10.3389/fvets.2021.718743 (PMC8450515; doi:10.3389/fvets.2021.718743)
Supplement: Supplementary file 1 [file Table_1.DOCX]

**Supplementary Table 1. The real-time RT-PCR primers used in the study.**

| **Primer names** | **Sequences (5′ to 3′)** |
| --- | --- |
| MCAD | Forward: 5'-TAACATACTCGTCACCCTTC-3' |
|  | Reverse: 5'-ATGCCTGTGATTCTTGCT-3' |
| CYP4 | Forward: 5’-GCAAACCATACCCAATCC-3’ |
|  | Reverse: 5'-TCCCAAGTGCCTTTCCTA-3' |
| PPARα | Forward: 5'-CTGTCGGGATGTCACACAATGC-3' |
|  | Reverse: 5'-TCTTTCAGGTCGTGTTCACAGGTAA-3' |
| CS | Forward: 5'-CGAATTTGAAAGATGTACTGAGC-3' |
|  | Reverse: 5'-CTTAGGCAGCATTTTCTGGC-3' |
| PK | Forward: 5'-CAGCCATGGCTGACACCTTC-3' |
|  | Reverse: 5'-GGATCAGATGCAAAGCTTTCTG-3' |
| GLUT4 | Forward: 5'-GATTCTGCTGCCCTTCTGTC-3' |
|  | Reverse: 5'-ATTGGACGCTCTCTCTCCAA-3' |
| G6PC | Forward: 5'-TGGACGGAAGCAATTTTTCA-3' |
|  | Reverse: 5'-GTCTCACAGGTGACAGGGAAC-3' |
| CPT-1α | Forward: 5'-TATGGTCAAGGTCTTCTCGGGTCG-3' |
|  | Reverse: 5'-AGTGCTGTCATGCGTTGGAAGTCTC-3' |
| FAS | Forward: 5'-TCGGAGACAATTCACCAAACC-3' |
|  | Reverse: 5'-AGCCATCCCACAGGAGAAACC-3' |
| ACC1 | Forward: 5'-TGCTGGATTATCTTGGCTTCA-3' |
|  | Reverse: 5'-CCCGTGGGAGTAGTTGCTGTA-3' |
| β-Action | Forward: 5'-CGTGGGCCGCCCTAGGCACCA-3' |
|  | Reverse: 5'-TTGGCCTTAGGGTTCAGGGGGG-3' |
| SDHA | Forward: 5'-CCTCGAATGCAGACGTACGA-3' |
|  | Reverse: 5'-CAACACCATAGGTCCGCACTT-3' |
| PGC-1α | Forward: 5'-CGGAAATCATATCCAACCAG-3' |
|  | Reverse: 5'-TGAGGACCGCTAGCAAGTTTG-3' |
